# Strengthening research capacity through the medical education partnership initiative: the Mozambique experience

**DOI:** 10.1186/1478-4491-11-62

**Published:** 2013-12-05

**Authors:** Emilia Virginia Noormahomed, Ana Olga Mocumbi, Michael Preziosi, Albertino Damasceno, Stephen Bickler, David M Smith, Carlos Funzamo, Eliah Aronoff-Spencer, Roberto Badaró, Francisco Mabila, David Bila, Alcido Nguenha, Virgilio Do Rosário, Constance A Benson, Robert T Schooley, Sam Patel, Luis Jorge Ferrão, Carla Carrilho

**Affiliations:** 1 Universidade Eduardo Mondlane, Av. Salvador Allende, 702, Caixa Postal 257, Maputo, Moçambique; 2 University of California, San Diego, 9500 Gilman Dr. MC 0679, La Jolla, San Diego, CA 92093, USA; 3 Universidade Lúrio, Reitoria, Bairro de Marrere, Rua 4250, Km 2,3, Caixa Postal 360, Nampula, Moçambique; 4 Instituto Nacional de Saúde, Av. Eduardo Mondlane, 1008, Caixa Postal 264, Maputo, Moçambique; 5 Hospital Central do Maputo, Av. Agostinho Neto, 1164, Maputo, Moçambique; 6 Ministério da Saúde, Av. Eduardo Mondlane, 1008, Caixa Postal 264, Maputo, Moçambique; 7 Universidade Federal da Bahia, Rua Augusto Viana S/N , 1° andar – Bairro Canela, CEP: 40110-060, Salvador, Bahia, Brasil; 8 MIHER- Mozambique Institute for Health Education and Research, Av. Salvador Allende, nº 745, Maputo, Mozambique; 9 Instituto de Higiene e Medicina Tropical, Universidade Nova de Lisboa, Rua Junqueira 100, Lisbon 1349-008, Portugal

**Keywords:** Research, Research capacity building in Mozambique, MEPI Mozambique

## Abstract

**Background:**

Since Mozambique’s independence, the major emphasis of its higher educational institutions has been on didactic education. Because of fiscal and human resource constraints, basic and applied research activities have been relatively modest in scope, and priorities have often been set primarily by external collaborators. These factors have compromised the scope and the relevance of locally conducted research and have limited the impact of Mozambique’s universities as major catalysts for national development.

**Case description:**

We developed a multi-institutional partnership to undertake a comprehensive analysis of the research environment at Mozambique’s major public universities to identify factors that have served as barriers to the development of a robust research enterprise. Based on this analysis, we developed a multifaceted plan to reduce the impact of these barriers and to enhance research capacity within Mozambique.

**Interventions:**

On the basis of our needs assessment, we have implemented a number of major initiatives within participating institutions to facilitate basic and applied research activities. These have included specialized training programmes, a reorganization of the research administration infrastructure, the development of multiple collaborative research projects that have emphasized local research priorities and a substantial investment in bioinformatics. We have established a research support centre that provides grant development and management services to Mozambique’s public universities and have developed an independent Institutional Review Board for the review of research involving human research subjects. Multiple research projects involving both communicable and non-communicable diseases have been developed and substantial external research support has been obtained to undertake these projects. A sizable investment in biomedical informatics has enhanced both connectivity and access to digital reference material. Active engagement with relevant entities within the Government of Mozambique has aligned institutional development with national priorities.

**Conclusions:**

Although multiple challenges remain, over the past 3 years significant progress has been made towards establishing conditions within which a broad range of basic, translational and clinical and public health research can be undertaken. Ongoing development of this research enterprise will enhance capacity to address critical locally relevant research questions and will leverage resources to accelerate the development of Mozambique’s national universities.

## Background

Following Mozambique’s independence in 1975 the Universidade de Lourenço Marques became the Universidade Eduardo Mondlane (UEM) [1], the only university for 8,233,000 citizens with a literacy rate of only 10.3% [2]. Most of UEM’s 2,500 students left Mozambique and none of the existing faculty were indigenous Mozambicans. Social sciences and economics were the first educational priorities, although medical, veterinary and health sciences and engineering were also designated as important. Although UEM fully recognized the importance of research for the creation of new knowledge, staff and infrastructure development, and for collaboration with external partners, the initial focus was on educational activities. Early faculty members had limited research skills and struggled to compete internationally for research support. Sporadically obtained funds supported research projects that were primarily planned by external collaborators, and not always aligned with locally defined research priorities. Non-governmental organizations (NGOs) supported graduate training or specific research programmes. Very little support was available to conduct essential, locally planned research projects or training.

Recognizing the importance of research for Mozambique’s development, the Government of Mozambique created the Ministry of Science and Technology (MIST) [3]. MIST is supported by the government and external donors including the World Bank, United Nations Development Programme and the Swedish International Development Cooperation Agency. Although extremely useful, MIST funds are not regularly available and have been of limited help in equipping laboratories for contemporary research and in sustainably supporting research teams.

**Figure 1:**
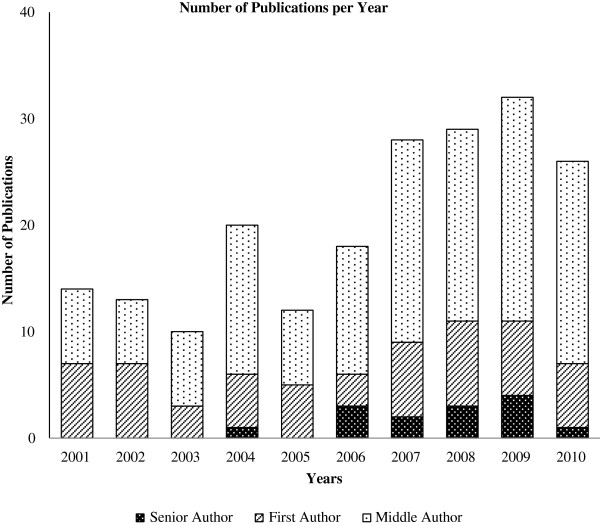
Number of peer-reviewed manuscripts published by the Universidade Eduardo Mondlane Faculty of Medicine and the Maputo Central Hospital from 2001 to 2010.

In Mozambique, medical research (heavily focused on HIV, malaria, tuberculosis and maternal and child health) is often conducted by NGOs in collaboration with public and private health institutions. Between 2001 and 2010, 202 peer-reviewed manuscripts were published by the 60 members of the UEM Faculty of Medicine (UEM-FoM) and the 140 physicians of its teaching hospital, Maputo Central Hospital (MCH) (Figure 1). Mozambicans were first authors of only 58 (29%) of these manuscripts, and, until recently, most were generated by a small number of research groups directed by senior specialists.

A 2011 study reported that less than 10% of African medical school faculty was involved in sponsored research [4]. Institutions in South Africa create 39% of the sub-Saharan medical literature, with first authorship dominated by researchers from American or British institutions [4,5]. A study conducted from 2001 to 2007, involving eight African universities that included UEM, showed that UEM had the lowest annual research publication rate per faculty member (0.03) while the University of Cape Town had the highest rate (1.14) [6].

In 2008, a partnership was formed between UEM and University of California, San Diego (UCSD) that was expanded under the Medical Education Partnership Initiative (MEPI) and has reinvigorated research development in Mozambique. Supported by the US Department of State through the President’s Emergency Plan For AIDS Relief and the National Institutes of Health (NIH), the UEM-UCSD MEPI seeks to: a) strengthen training of physicians; b) increase the capacity for locally driven, multidisciplinary research; c) strengthen the informatics infrastructure; and d) recruit and retain qualified medical faculty [7]. We report here our experience with the research capacity-building aspects of the UEM-UCSD MEPI. Although the MEPI was established as a bilateral partnership between UEM and UCSD, it has served as a framework to leverage, integrate and strengthen relationships with other institutions in Africa, Europe and South America.

To create a highly competitive group of researchers at UEM and allied institutions, and to enhance the long-term sustainability of the MEPI investment by expanding the number and supporting retention of qualified faculty members, we developed a plan to improve teaching quality, health care delivery systems, and research capabilities at UEM and two new public universities (Unilurio and Unizambeze) in two geographically critical areas of northern Mozambique. These efforts also seek to stimulate south-south collaborations through faculty exchange, jointly conducted research and sharing of training infrastructure. We describe here the strategies adopted to strengthen research capacity in Mozambique’s public universities under the UEM-UCSD partnership.

## Case description

### Needs assessment methodology

**Figure 2:**
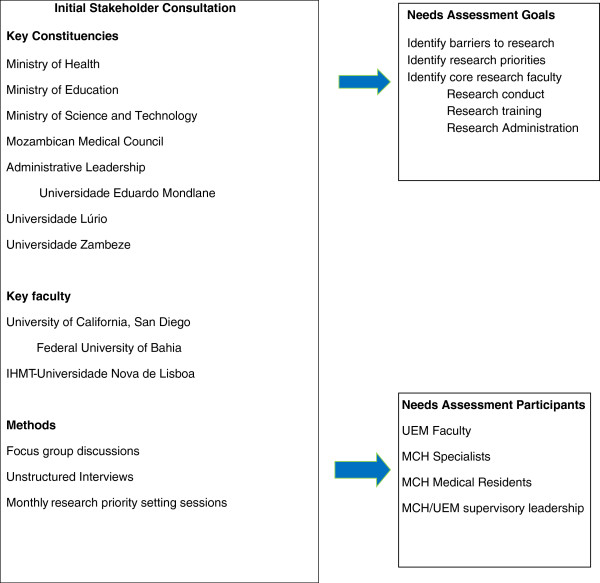
**Initial needs assessment: participants and methodologies employed.** IHMT, Instituto de Higiene e Medicina Tropical; MCH, Maputo Central Hospital; UEM, Universidade Eduardo Mondlane.

After consultation with the Ministries of Health (MOH) and Education (MOE), the MIST, the Mozambican Medical Council (MMC), and the Vice Chancellors of UEM, Unilurio and Unizambeze, a core group of faculty from UEM, UCSD, the Federal University of Bahia (UFBA; Brazil) and the Instituto de Higiene e Medicina Tropical-Universidade Nova de Lisboa (IHMT-UL; Portugal) identified obstacles to conducting research in Mozambique, defined initial research priorities, established robust teams of mentors and mentees, and developed a roadmap for building operational, epidemiologic, translational and clinical research capacity and infrastructure within Mozambican partner institutions. Broad input was obtained through focus group discussions and unstructured interviews with critical stakeholders (medical postgraduate trainees, supervisors, specialists, faculty). UCSD and other visiting faculty were assigned mentorship roles to enhance local capacity in key areas including grant and manuscript writing, research project design and advanced clinical teaching skills (Figure 2).

### Findings

Several significant barriers and gaps that contributed to the underdevelopment of research in Mozambique were identified.

#### Institutional environment

Research in Mozambique has historically been undervalued and, thus, conducted by a small minority of the faculty. Because of limitations in institutional salaries and ill-defined mechanisms to route salary support from externally funded research projects, faculty had to dedicate substantial efforts to the private practice of medicine and other extra-institutional activities to meet living expenses. These constraints affected senior faculty making them less available to mentor younger research faculty. The fiscal challenges have been compounded by the lack of professional recognition and career development opportunities within Mozambican universities for researchers and by internal migration to NGOs due to scale-up of HIV, tuberculosis and malaria programmes offering salaries substantially higher than those within universities. Among medical doctors graduating between 1980 and 2006 in Mozambique, 25% left the public sector by July 2010, and of those 62.4% were employed within the country [8].

#### Human resource limitations

At the time the UEM-UCSD MEPI began, the UEM FoM consisted of 181 faculty members (60 full-time and 121 part-time) and MCH had a total of 300 staff physicians. Of these only about 100 had received postgraduate medical training. Few were engaged in externally funded medical or scientific research, and those that were limited their activities to less than 8 hours per week. In addition to the limited human resources available, contemporary research methodological skills are lacking within the university-based Mozambican research community. Inexperience in grant writing has made it difficult for Mozambican scientists to compete successfully for peer reviewed international funding. As a Lusophone country, English language skills among Mozambican scientists are imperfect. This limits both access and contributions to the heavily Anglophone global scientific literature.

#### Research infrastructure limitations

Within Mozambican universities there was and is a dearth of well-equipped laboratories or contemporary research equipment. For example, only one laboratory in the FoM was equipped to perform polymerase chain reaction experiments. Internet bandwidth was limited and variably available in a few offices. Wireless access to the internet was not available in either the FoM or MCH. The FoM library maintained primarily a print collection – much of which was outdated – and digital resources were extremely inadequate.

#### Lack of financial resources, grants administration and administrative management policies

The overall shortage, sporadic availability, and inefficient administration of research funding have severely limited research development. Inconsistent administrative policies and lack of fiscal management infrastructure have been particularly damaging. Such capacity is fundamental to attract and properly manage research activities. The administrative structure of UEM and other public universities is based on state public sector management principles that have not evolved to incorporate contemporary management principles of investigator-initiated externally funded research. This negatively affects timely and accountable preparation and submission of research proposals and poses further challenges when grant funds are awarded.

Salaries within Mozambican higher educational institutions are set at levels that reflect the direct teaching responsibilities of university faculty. Faculty is expected to generate the remainder of their income from external activities. Consequently, faculty may teach for several hours in the morning for a base teaching salary, with the expectation that they will work for external agencies or in private medical practices for the rest of the day to generate the remainder of their income. If faculty generate additional funds for research activities, it is difficult to route these funds through university administrative structures to replace income from external activities since funding agencies may not recognize that the base teaching salary is not intended as a full-time salary. Thus, non-research income generating activities outside the university take priority over research as faculty scramble to meet living expenses.

#### Limited access to scientific literature

Access to contemporary medical and scientific literature is constrained by inadequate investment in library resources. Paper journal collections are extremely sparse. Although Mozambican faculty has free digital access to medical and scientific literature through the HINARI initiative, some are not aware of this resource. Digital access is further compromised by inadequate internet bandwidth and access to data terminals, and incomplete awareness of how to access digital resources at affordable costs.

#### Human research subject protection and ethical review

Because all human subject research was reviewed by a single National Ethics Committee, the process was slow and unpredictable. This stifled the motivation of the research community, and discouraged those contemplating research careers.

### Interventions

**Figure 3:**
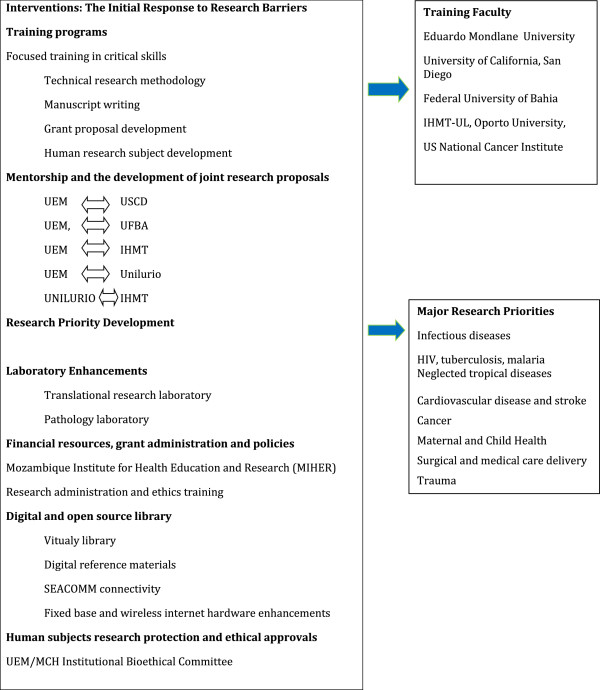
**Interventions: the initial response to research barriers.** IHMT-UL, Instituto de Higiene e Medicina Tropical Universidade Nova de Lisboa; MCH, Maputo Central Hospital; UEM, Universidade Eduardo Mondlane; UFBA, Federal University of Bahia; USCD, University of California, San Diego.

Our comprehensive assessment of the barriers to research productivity within Mozambique’s public universities resulted in several actions (Figure 3).

#### Training programmes

To address low productivity due to lack of technical competencies, training programmes in research methods, grant and manuscript writing and human research subject protection were organized for faculty and postgraduate trainees and regularly presented at UEM and Unilurio by UEM faculty and international partners. These programmes have also been formally integrated into the MEPI-supported postgraduate training programme in conjunction with the MOH, the Medical Council and the National Postgraduate Commission. Thirty-six postgraduate trainees and specialists from the UEM-FoM and MCH attended and received certificates from an online course on Human Research Subjects Protection developed by the National Cancer Institute of the US NIH. MEPI facilitators enhanced active participation. Four research methods courses were successfully completed by 82 students. Forty-four candidates participated in a course on manuscript writing. A grant writing workshop was completed by 30 UEM/MCH faculty and postgraduate trainees. Courses were conducted by senior UEM faculty in conjunction with faculty from our partner institutions. Each course required formal registration, relief of ongoing duties and certificates were awarded for successful completion. The research methods and grant writing courses are being adapted for a distance learning platform to extend the reach of these programmes with marginal additional investments. Finally, components of the UCSD Clinical Research Enhancement with Supplemental Training programme were implemented for junior faculty and postgraduate trainees from UEM/MCH and are being incorporated into a UEM-based Master’s Degree programme currently in development.

#### Mentorship and the development of joint-research proposals

Mentors from the Clinical Microbiology Laboratory and the Departments of Internal Medicine, Pediatrics, Surgery, Orthopedics and Gynecology/Obstetrics of MCH and the Departments of Microbiology and Pathology of UEM and collaborating institutions were also identified and paired with mentees to jointly develop research topics for grant writing. This was undertaken to augment didactic training in grant preparation to further refine local research priorities and to acquire external support for these research priorities. The communicable and non-communicable diseases identified as initial priority areas for research included HIV, malaria, human papillomavirus and associated malignancies, tuberculosis, cardiovascular diseases, cancer, child health, trauma, and neglected infectious diseases such as cysticercosis, toxoplasmosis and schistosomiasis. Monthly sessions were established for postgraduate trainees, faculty, and investigators to present, discuss, and develop research proposals and protocols, and enhance research proficiency.

Since beginning these efforts, US$789,123 has been awarded to 13 different research projects following competitive reviews by a number of American and European granting agencies (Table 1). As mentors and mentees have begun to see the impact of these grants in their work environment, strong motivation has been provided for others to initiate research careers. As a result, other research projects are under review or development (Table 2). The new support has also allowed for stipends to key faculty and students to replace extra-institutional activities with protected research or clinical training time.

**Table 1:** Funded research projects

**Funded projects**	**Funding source and amount (US$)**
Immunogenetic variation in schistosomiasis and HIV co-infection	UCSD CFAR ($40,000)
Etiology of bloodstream infections at MCH	UCSD CFAR ($40,000)
Multiplex diagnostic system for the diagnosis of infectious diseases and quantification of CD4+ cells	mBio diagnostics ($20,000)
Interactions between HIV-1 and *Cysticercus cellulose* in Beira, Mozambique	UCSD CFAR ($40,000)
Etiologic role of human papillomavirus in conjunctival squamous cell carcinoma in Mozambique	UCSD CFAR/CC NCI ($51,523)
Hepatocellular carcinoma in patients with HIV and HBV co-infection in Mozambique	UCSD CFAR/CC NCI ($51,411)
Pooled nucleic acid testing to identify virologic failure during antiretroviral therapy	NIH ($378,189)
Study of genetic diversity and primary resistance to lamivudine for hepatitis B virus in individuals coinfected with HIV in the area of health Mavalane	UL/INS ($22,750)
Study of the frequency of occult hepatitis B in HIV co infection in the area of Health Mavalane	UL/INS ($22,750)
Prevalence and incidence of latent tuberculosis among workers in the MCH - Health and usefulness of Interferon-gamma release assays to detect latent tuberculosis infection	UCSD CFAR ($40,000)
Cardiovascular risk and disease in HIV-infected patients in Mozambique	UCSD CFAR ($40,000)
Characterization of *Salmonella* spp in patients admitted to the department of medicine	UCSD CFAR ($40,000)
Frequency of cryptococal antigenemia in HIV positive patients without antiretroviral treatment	RU-DoM-MCH ($2,500)
**Other projects to which funding has been committed**	
Creation of a joint institutional ethics committee of Faculty of Medicine of the UEM and MCH	EDCTP ($58,500)
Cure club foot project	Cure international ($123,666)

EDCTP, European Developing Countries Clinical Trials Partnership; INS, Instituto Nacional de Saude; NCI, NIH National Institute of Cancer NIH, United States National Institute of Health; RU-DoM-MCH, Research unit of the Department of Medicine, Maputo Central Hospital; UCSD CFAR, University of California San Diego Center for AIDS Research; UEM, Universidade Eduardo Mondlane; UL, University of Lisbon.

**Table 2:** Research projects under review or in development

**Project title**	**Current status**
Epidemiology and registry of rheumatic heart disease	In development
Epidemiology and registry of pulmonary hypertension	In development
Causes of sudden and unexpected deaths in the medico-legal Department of MCH	Under review
Determinants of low birth weight in HIV positive women in rural Mozambique	Under review
The role of oxidative stress and cell cycle dysfunction in growth failure of Mozambican children	Under review
Pilot study on neurocysticercosis and HIV/AIDS in epileptic patients from Nampula	Under review
Prevention of paediatric burns in a rural area of Mozambique	Under review
MCH, Maputo Central Hospital	Current status

Formal partnerships intended to promote south to south collaboration have also been initiated. These include joint proposals with collaborators from UFBA, the University of KwaZulu Natal, the University of Cape Town, the University of Zimbabwe, the University of Nairobi and the Nestlé Nutrition Institute Africa.

#### Laboratory enhancements

Laboratory infrastructure enhancements and new equipment purchases were made in both the Departments of Microbiology and Pathology. Renovation of these laboratories and additional training of the technical staff converted them into specialized Infectious Diseases and Pathology Laboratory Centres. Core technical areas were identified for further development in molecular biology, clinical diagnostics, immunochemistry, cytopathology and genetics, and equipment was purchased with support from the Gilead Foundation (Foster City, CA, USA), the James B. Pendleton Trust (Belleview, WA, USA), MBio Diagnostics (Boulder, CO, USA) and the MEPI programme. Faculty members from UCSD, UFBA, IHMT-UL and UEM were exchanged for training purposes and to form collaborative research groups. Mozambican laboratory technicians were trained by collaborators in Mozambique and in the UCSD Infectious Disease and Pathology laboratories, and supplemented with ongoing training and mentorship of laboratory personnel. Thus, state-of-the-art laboratory facilities are now available for training and for the conduct of research.

#### Financial resources, grant administration and policies

To address the limitations in administrative and fiscal management, a research support centre (the Mozambique Institute for Health Education and Research (MIHER); http://www.miher.org) was created to provide administrative and fiscal management support to the scientific community of UEM and other public universities within Mozambique and to identify funding agencies that might be interested in supporting research activities.

Research administration training has been initiated. Two UEM/MIHER staff completed research administration courses in Biomedical and Behavioral Sciences organized by the US National Institute of Child Health and Development and an additional workshop on grants management organized by the US National Institute of Allergy and Infectious Diseases. These staff development courses provided comprehensive training in grant and financial management and research ethics administration, and positioned MIHER to provide administrative and fiscal support for faculty at UEM and Mozambique’s two new public universities.

#### Digital and open source library

After carefully assessing systematic deficiencies in reference materials, we elected to invest primarily in digital reference resources. We established a Virtual Library to be shared by UEM, Unilurio, and Unizambeze faculty, staff and students. Regular training sessions for use of online and computing resources have been established by UEM’s Centre for Informatics. Key content is available through HINARI, Medicine Central (Kind donation of Dr Bill Detmer, Unbound Medicine) and Elsevier Clinical Key (available at a reduced cost from Elsevier).

#### Human subjects research protection and ethical approvals

To accelerate human subject research review, members of the MEPI, the UEM FoM, MCH and UCSD were awarded a grant by the European Developing Countries Clinical Trials Partnership to create a joint Institutional Review Board (IRB) for the FoM and MCH. This IRB is now active and will progressively supplant the National Ethics Committee as the IRB of record for biomedical and behavioural research conducted at UEM and MCH. This will substantially shorten review timelines and greatly facilitate human subject research within our institution.

### Outcomes

Although a number of our initiatives are only now gaining momentum, important achievements have been accomplished.

#### Research proposals and funding

During the first 28 months of the MEPI programme, 30 new research projects involving mentors and collaborators from UEM, UCSD, UFBA and IHMT-UL were proposed in priority areas. Thirteen of these have received external funding and funding has been committed to two additional projects. Another seven projects are under review and two of these have been selected by sponsoring agencies for full proposal development. Other proposals are still in process. In addition, two manuscripts are in press, five others are under review and an additional five are in preparation.

#### Faculty development and retention

A multidisciplinary group of faculty from UEM, UCSD, UFBA and the IHMT-UL has been created that will provide ongoing mentoring of operational, epidemiologic/public health, translational and clinical research capacity development within Mozambican partner institutions. A motivated and dedicated group of mentees has emerged to carry the effort forward. Rather than being provided as external “enrichment” courses, research training courses have been developed as recurring activities led by Mozambican faculty and have been integrated as obligatory components of postgraduate medical training by the MMC. By aligning these activities within the university with recently re-energized national regulatory and governmental entities such as the MMC and the MOH, they have become expectations of trainees and have attracted local investments to ensure sustainability. Adding research capacity as a career path enhances faculty retention and contributes to sustainability of the programmes and projects that have been started.

#### Research support infrastructure

Additional sustainability has been assured by the transformation of the former Parasitology and Pathology Laboratories into institutional laboratory “Centres of Excellence” in which numerous research activities can be supported on an ongoing basis. Intellectual and fiscal sustainability will be further ensured by the infrastructure renovation and provision of contemporary equipment, complemented by training of technical personnel in current cell and molecular biological techniques.

The establishment of MIHER as the first research support centre within Mozambique creates a mechanism to collectively support researchers and junior faculty throughout the country in project design, grant preparation and submission, and administrative and fiscal management of externally funded research activities. This has amplified research capacity at UEM and has positioned it to leverage support for parallel activities in Mozambique’s two new medical schools. This centre will also support collaborative research efforts among Mozambican researchers, regionally and internationally, and will identify additional sources of financial support, which is one of the essential metrics for success of the MEPI in Mozambique. The joint IRB for the UEM FoM and MCH will also substantially streamline research implementation.

## Discussion

Although many factors have contributed to the early successes of these efforts, we believe the key to our progress has been the structure of our partnership. Working with our international partners we first identified the major barriers to conducting medical and scientific research within Mozambique. We then worked with our partners to develop locally applicable approaches to overcome them. This approach is, perhaps, the inverse of “traditional” collaborations in which an international entity with externally defined research priorities seeks local support to address their priorities. Although these projects may have substantial value (both globally and locally), they are often dependent on the ongoing interests of the external collaborator and, therefore, fail to develop sustainable, broad-based research capacity in the country. Our MEPI partnership relies heavily on local priority setting and emphasizes the development of local expertise to sustain the infrastructure for research and training. Faculty and laboratory technicians from the collaborating universities have reciprocally visited to jointly design research grants, to provide training in research methods and grant and scientific writing, and to initiate research projects. The Mozambican MEPI aims were jointly designed by researchers from UEM and UCSD according to their best understanding of Mozambique’s needs, while remaining aligned with policies and priorities of the Mozambican government and key local institutions including the MOH, MOE, the MMC, MIST, UEM, Unilurio and Unizambeze, thus assuring broad ownership by local stakeholders. This innovative approach assures future sustainability as the activities are undertaken by participants and are not viewed as being imposed with externally defined goals that may negatively impact research productivity and relevance [9]. Research will have more impact if local researchers are enabled to take leadership roles and define research priorities according to the country’s needs. The channelling of research and development funds through universities and affiliated research support centres also enhances sustainability of public institutions and combats internal “brain drain” to the private sector and NGOs.

### Moving forward

There is significant potential for research collaboration in multiple areas of infectious and non-communicable diseases. Although our initial aim was to enhance research capacity and develop new knowledge about pressing local health problems, we also aligned research activities to support policies and interventions of the MOH, MOE and MIST to provide additional capability to evaluate and monitor other national health, education and research priorities.

Further steps to stimulate clinical and operational research will be undertaken by strengthening the existing relationships among UEM, UCSD, UFBA, IHMT-UL, Unilurio and Unizambeze, and other partners for study design, grant applications, publications and research methods and ethics courses. Joint planning, mentoring and exchange of faculty and research staff create synergies to better leverage limited resources. Whenever possible, equipment, human and other resources should be shared among local, regional and international institutions. Publishing findings in scientific literature is critical to sustaining the goals defined under our MEPI programme. A system of differential rewards for those who publish should be created within universities. Building additional skills through ongoing courses on grant and scientific writing, and exposure to new research methods and research ethics remains critically important. Further enhancement of the information technology infrastructure is also crucial for the conduct of contemporary research. Additional motivating factors, such as tuition waivers/remissions and fellowships for career development would also enhance faculty retention.

A network of researchers, politicians and policy makers should be created and discussion forums established to facilitate exchange of ideas so that politicians can allocate resources according to where they are most needed. The creation of modules for research training at undergraduate universities should be developed to form the basis for future research careers. Scholarships directed at generating a critical mass of researchers and resources to facilitate participation in international conferences should be made available. Additional educational alliances and research collaborations with Brazil and other Lusophone countries would also be beneficial.

Government, donors and finance agencies should establish appropriate salaries, including stipends for protected time, for internationally competitive researchers, and should create additional career posts and fellowships for postdoctoral research. To ensure the sustainability of research investments in countries such as Mozambique, we suggest research sponsors be sensitive and receptive to the financial and human resource constraints of sub-Saharan African universities and align their granting policies with the realities of local politics, bureaucracy and taxation rules. Finally, as the essential benefits of locally planned, university-based research activities are recognized by key national institutions such as the MOH and the MOE, and as Mozambique’s economy improves, additional governmental investment in locally supported peer-reviewed research should be made. This capacity could be provided either through MIST or by developing competitive extramural funding mechanisms through the MOH’s Instituto Nacional de Saúde.

Although much progress is already apparent, the timeline to achieve the overall goals of our collaboration will be measured in decades rather than months or years. Patience will be required but the increasing sense of optimism and local investment in a successful research enterprise represents a sea change within Mozambique. We believe this will have far reaching consequences that extend to improved health, accelerated economic development and an ongoing investment of local resources to sustain these gains as their multifaceted impacts are increasingly appreciated. Since many of the same obstacles have been encountered by many Sub-Saharan African countries [9], we hope that adapting some of the same strategies can help other countries build sustainable research efforts that can help to increase capacity across the region.

## Conclusions

Strengthening research capacity in universities located in resource limited settings is essential to educational, cultural and economic development. UEM, Mozambique’s oldest national university, has made major strides in developing its research capacity through a unique partnership with UCSD and several additional universities in Brazil, Portugal and South Africa. By focusing locally developed priorities to enhance faculty development, laboratory capacity and the administrative and informatics infrastructure, this partnership has positioned UEM to expand Mozambique’s indigenous research footprint and to serve as the focal point for developing similar capacity in the two newly established public universities in central and northern Mozambique. Multilateral partnerships focused on development of research capacity in resource limited settings can have a major impact on the functionality and vitality of academic institutions and on national development.

## Abbreviations

IHMT-UL: Instituto de Higiene e Medicina Tropical Universidade Nova de Lisboa; IRB: Institutional Review Board; MCH: Maputo Central Hospital; MEPI: Medical Education Partnership Initiative; MIHER: Mozambique Institute for Health Education and Research; MIST: Ministry of Science and Technology; MMC: Mozambican Medical Council; MOE: Ministry of Education; MOH: Ministry of Health; NGO: Non Governmental Organization; NIH: National Institutes of Health; UCSD: University of California, San Diego; UEM: Universidade Eduardo Mondlane; UEM-FoM: Universidade Eduardo Mondlane Faculty of Medicine; UFBA: Federal University of Bahia.

## Competing interests

The authors declare that they have no competing interests.

## Authors’ contributions

EVN and RTS conceived the original proposal and strategy to develop research capacity and are co-directors of the programme and responsible for the overall implementation. AOM, CC, AD, SP, MP, EA-S, CAB, DMS, and SB were responsible for implementing the strategy and contributed with content and editing to the current manuscript. RB and AN co-directed the creation of the research support centre, and contributed content and editing to the current manuscript. LJF and VDR co-directed the implementation of capacity building strategy to develop research, (especially at Lúrio University) and contributed with content and editing of the manuscript. FM and DB co-formulated and implemented the bioinformatics strategy and implementation. All authors read and approved the final manuscript.
